# Effects of 5 Years of Aerobic Exercise on Sarcopenia in Older Adults—Secondary Outcomes of the Generation 100 Study

**DOI:** 10.1002/jcsm.70279

**Published:** 2026-04-05

**Authors:** Guro Birgitte Stene, Maria Jose Lequerica, Dorthe Stensvold, Trude Rakel Balstad, Stian Lydersen, Ulrik Wisløff, Jorunn Lægdheim Helbostad

**Affiliations:** ^1^ Department of Neuromedicine and Movement Science, Faculty of Medicine and Health Science The Norwegian University of Technology and Science (NTNU) Trondheim Norway; ^2^ Centre of Crisis Psychology, Faculty of Psychology University of Bergen Bergen Norway; ^3^ Department of Circulation and Medical Imaging, Faculty of Medicine and Health Science The Norwegian University of Technology and Science (NTNU) Trondheim Norway; ^4^ Clinic of Cardiology and Cardiothoracic Surgery St. Olavs Hospital Trondheim Norway; ^5^ Department of Clinical and Molecular Medicine, Faculty of Medicine and Health Science The Norwegian University of Technology and Science (NTNU) Trondheim Norway; ^6^ Cancer Clinic, St. Olavs Hospital Trondheim University Hospital Trondheim Norway; ^7^ Department of Mental Health, Faculty of Medicine and Health Science The Norwegian University of Technology and Science (NTNU) Trondheim Norway; ^8^ Department of Geriatrics, Department of Medicine, St. Olavs Hospital Trondheim University Hospital Trondheim Norway

**Keywords:** aerobic exercise, gait speed, muscle strength, older adults, prevention, sarcopenia

## Abstract

**Background:**

Early prevention of sarcopenia through exercise is vital to health, independence and mortality risk in older adults; however, there is a scarcity of evidence concerning the long‐term effects of aerobic exercise alone. We tested the primary hypothesis that a 5‐year decline in key defining components of sarcopenia is less in an aerobic exercise group compared with a control group. Secondly, we tested the same hypothesis using a model of ‘clinically defined sarcopenia’.

**Methods:**

Norwegian community dwelling older adults (*n* = 1567; aged 70–75 years at inclusion) participated in the Generation 100 Study. They were randomized in a 1:1:2 ratio—stratified by sex and cohabitation status to either moderate intensity continuous training (MICT, *n* = 387) or high intensity interval training (HIIT, *n* = 400), or to a control group following the Norwegian physical activity guidelines (CON, *n* = 780). Key defining components of sarcopenia were grip strength (kilograms), skeletal muscle index (kg/m^2^) and gait speed (m/s). Clinically defined sarcopenia was based on the European Working Group on Sarcopenia in Older People and recent normative data for the Norwegian population. An intention to treat linear mixed model and an ordinal logistic regression mixed model were used to examine the effects of aerobic exercise on sarcopenia on the 1, 3 and 5 years follow‐up. *p* values for the main hypothesis were adjusted using the Benjamini–Hochberg correction.

**Results:**

Mean age across groups was 72 years of age, including ~50% women, ~50% with higher education, 86%–90% with good or very good health and 73%–76% considered physically active. Compared with CON, grip strength declined less for HIIT at Year 1 (estimated difference of 0.98 kg; *p* < 0.001) and Year 3 (estimated difference of 0.03 kg; *p* = 0.016). At Year 3, HIIT increased their gait speed by 0.01 m/s (estimated difference between HIIT vs. CON of 0.03 m/s; *p* = 0.016). At Year 5, all groups declined from baseline (CON −0.07; MICT −0.10 and HIIT −0.04 m/s). Participants determined as having ‘clinically defined sarcopenia’ at baseline, Years 3 and 5 were: 22%, 58% and 63% (CON); 20%, 55% and 67% (MICT) and 19%, 50% and 56% (HIIT). Odds for developing clinically defined sarcopenia were significantly lower for HIIT (OR = 0.51; *p* = 0.018 at Year 3; OR = 0.47; *p* = 0.009 at Year 5) as compared with CON. No effects were found for MICT.

**Conclusions:**

The study demonstrates that in addition to having a protective cardiovascular effect, HIIT also has a beneficial effect on muscle strength and physical performance in a relatively healthy population of older adults.

## 1 Introduction

Sarcopenia is a progressive, age‐related skeletal muscle disorder marked by declining muscle mass and strength and/or physical performance [1]. It is associated with adverse health outcomes [1, 2], including increased risk of falls and fractures [3, 4], dependency in activities of daily living [5], mobility impairment [6], diminished quality of life [7] and premature death [8].

The European Working Group on Sarcopenia in Older People (EWGSOP2) defines low muscle strength as the primary criterion for ‘probable sarcopenia’. The diagnosis (‘sarcopenia’) is confirmed by low muscle mass, and severity is graded by impaired physical performance (‘severe sarcopenia’) [1]. A meta‐analysis incorporating diverse definitions and thresholds across Europe, the United States of America (USA) and Asia estimates sarcopenia prevalence in adults aged ≥ 60 years to range from 10% to 27% [9]. So far, prevalence estimates from Scandinavian countries, including Norway, are lacking.

Despite its inclusion in the International Classification of Diseases (ICD M62.84) in 2016 [10], sarcopenia is still not a universally accepted clinical definition [11], and the condition remains underdiagnosed [12], which might have contributed to delayed recognition and suboptimal management [13]. Importantly, we do know that sarcopenia might start subtly beyond the age of 50 and develop gradually unless counteracted [1]. Strategies for early detection and prevention of sarcopenia are critical to enable older adults to keep functionally fit, maintain independence in activities of daily living as long as possible and enhance quality of life and longevity [14]. Physical exercise is well established as a preventive strategy against sarcopenia [15] and is recommended as first‐line therapy [16]. Resistance training is particularly effective in mitigating declines in muscle mass, muscle strength and physical performance, as demonstrated in well‐functioning older adults [17, 18] and in individuals who are presarcopenic [19] or have sarcopenia [20]. Systematic reviews [21, 22, 23, 24] further support the benefits of multimodal interventions, including aerobic exercise.

The effect of aerobic exercise alone has been sparsely assessed. A cross‐sectional study in older adults found regular resistance training but not aerobic training alone to improve muscle mass and strength [25], while a treadmill intervention study in obese older adults found that 24 weeks of aerobic training (walking three times a week for 45 min at 70% of heart rate reserve) resulted in improved muscle quality (51% increase in basal myofibrillar protein synthesis; 65% increase in capillary density) and strength (15% increase in quadriceps strength) [26]. The latter finding is consistent with an earlier study indicating that aerobic exercise may preserve muscle function and attenuate sarcopenia progression [27]. Importantly, aerobic exercise also has clear cardioprotective benefits [28] and enhances physical function and quality of life in older adults [25].

To investigate the role of aerobic exercise in early prevention of sarcopenia, we analysed secondary outcomes from the Generation 100 Study—a 5‐year randomized controlled trial involving adults aged ≥ 70 years [29]. Our primary hypothesis was that declines in muscle mass, strength and physical performance in relatively healthy older adults would be attenuated in participants undergoing 5 years of moderate‐intensity continuous training (MICT) or high‐intensity interval training (HIIT) compared with a control group following the Norwegian physical activity guidelines (CON).

We further modelled clinically defined sarcopenia using EWGSOP2 criteria [1], adapted to regional normative data from healthy young Norwegians [30, 31, 32]. Our secondary hypothesis was that participating in MICT and HIIT would prevent the development of ‘clinically defined sarcopenia’ from baseline to 3 and 5 years, relative to the CON group.

## 2 Methods

### 2.1 Study Design, Setting and Participants

This study uses a longitudinal design over 5 years for a secondary analysis of the data from the Generation 100 Study [28, 29]. In 2012, all inhabitants of Trondheim, Norway, aged 70–77 years (born 1 January 1936 to 31 December 1942) were invited to participate in this study. Participants with serious heart conditions, diagnosed dementia, cancer, chronic communicable infectious diseases, illness or disabilities that precluded exercise, or participation in other exercise trials, were not eligible in the study as previously reported [28, 29].

**FIGURE 1:**
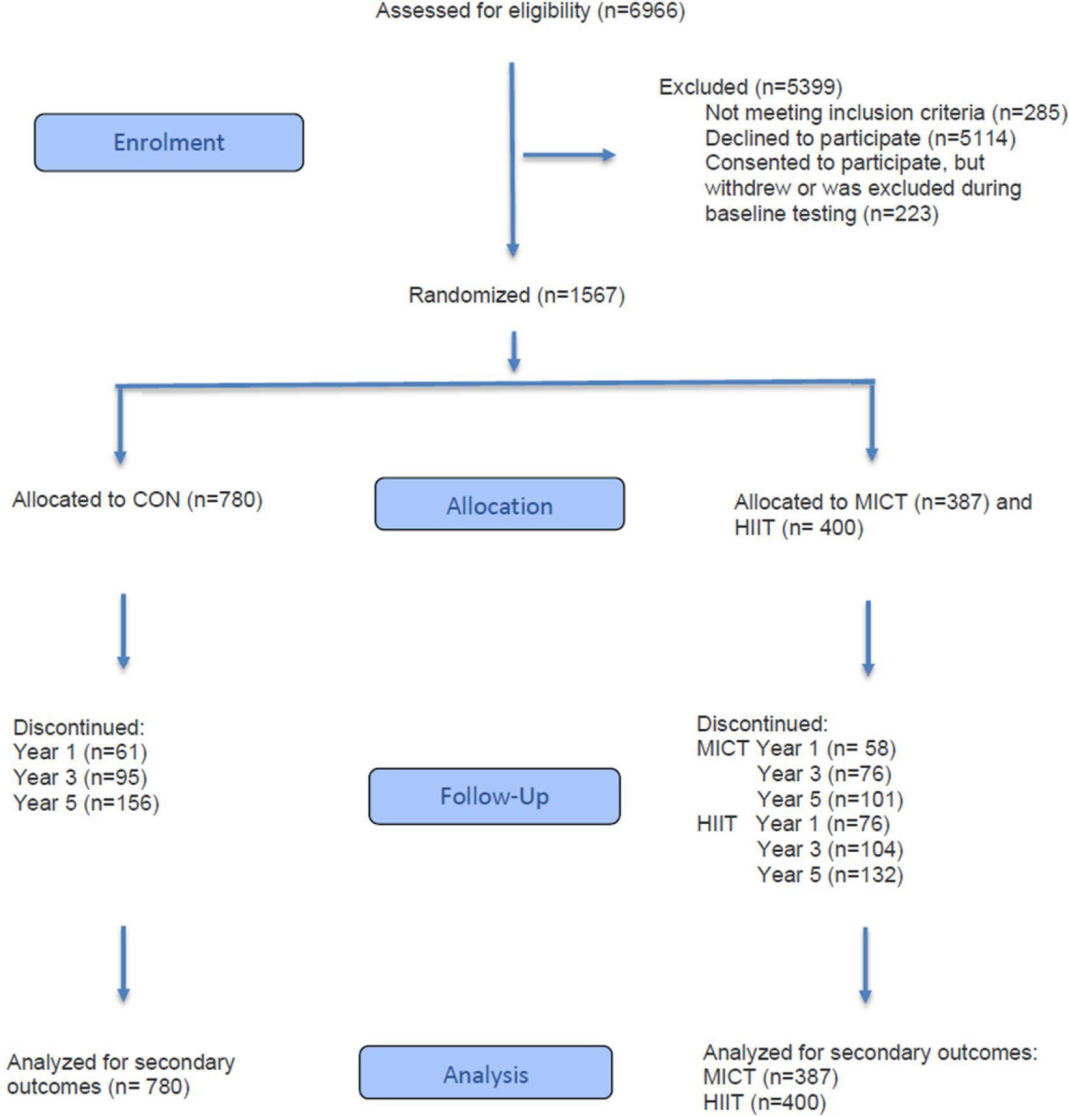
CONSORT flow diagram of participants in the study. MICT = moderate‐intensity continuous training. HIIT = high‐intensity interval training. CON = control group following national physical activity guidelines.

Of 6966 individuals screened for eligibility (Figure 1), 1567 were randomized in a 1:1:2 ratio—stratified by sex and cohabitation status (living alone vs. with someone)—to either *moderate‐intensity continuous training* (MICT; *n* = 387) or *high‐intensity interval training* (HIIT; *n* = 400), or to a group following the Norwegian physical activity guidelines (CON; *n* = 780). Participants in the same cohabiting pair were allocated to the same group. Participants were assessed at baseline (conducted in the period August 2012 to June 2013) and follow‐up assessments after 1, 3 and 5 years.

The study was approved by the Regional Ethics Committee before study onset (REK2012/381 B) and registered in ClinicalTrials.gov in August 2012 (NCT01666340).

### 2.2 The Interventions and Adherence to Exercise Prescription

Details of the interventions have been reported previously [28, 29]. In brief, participants in the MICT group completed 50 min of continuous moderate‐intensity exercise (70% of peak heart rate). The HIIT group performed a light 10‐min warm‐up, followed by walking or running uphill in four 4‐min intervals (85%–95% of peak heart rate) interspersed by 3‐min active breaks (60%–70% of peak heart rate), over a total duration of 38 min at each exercise session. Both groups were instructed to exercise twice weekly and had the opportunity to attend sessions led by a trained instructor in an outdoor walking environment. In addition, both HIIT and MICT participants met every sixth week for a supervised spinning session (ergometer cycling) to monitor heart rate and exercise intensity. The CON group was instructed to follow the current Norwegian physical activity guidelines, which at that time was 30 min of moderate‐ or high‐level physical activity 7 days a week [33]. To support long‐term adherence over the 5‐year intervention period, all participants were invited to annual meetings that provided general updates and information about the study.

A validated questionnaire on exercise frequency, intensity and duration was used to assess adherence to exercise at 1, 3 and 5 years follow‐up [34]. Not adhering to exercise was defined as having performed less than 50% of the prescribed training sessions over the 5 years [29]. In the Generation 100 Study, adherence to exercise prescription at Years 1, 3 and 5, respectively, was 50%, 47% and 47% for the HIIT group, 63%, 55% and 51% in the MICT group and in the CON group, 78%, 70% and 69% adhered to following the Norwegian physical activity guidelines [28].

### 2.3 Assessment of Baseline Characteristics and Outcomes

Baseline characteristics were assessed through clinical examination and questionnaires as previously described [29]. In this study, we included age, sex, body weight, height and body mass index (kg/m^2^). Self‐reported health was assessed by one item from the SF‐8 Health Survey (How will you, in general, consider your health during the last week?) graded as ‘Excellent, very good, good, poor or very poor’. Items from questionnaires used in The Trøndelag Health Study (The HUNT Study) in Norway [35] included: education (‘What is your highest level of education?’) and living status (‘Do you live alone or with spouse/partner/other?’).

Key defining components of sarcopenia included skeletal muscle mass, muscle strength, and physical performance. Total skeletal muscle mass (in kilograms) was assessed by bioimpedance, BIA (InBody 720, InBody Co. Ltd., Seoul, Korea). Gender‐specific skeletal muscle indexes (SMI) were calculated separately for men and women: skeletal muscle mass (kilograms, kg) = 0.244 × weight (kg) + 7.8 × height (meters, m) − 0.098 × age + 6.6 for men, and skeletal muscle mass (kg) = 0.193 × weight (kg) + 5.5 × height (m) − 0.244 × age + 2.9 [36] for women. Muscle grip strength in the dominant hand was assessed by a JAMAR Hydraulic Hand Dynamometer (Lafayette Instrument Company, USA) [37] with average grip strength (kilograms) calculated over three trials. Physical performance was assessed by gait speed derived from the Gaitrite (at baseline) or ZENO (3 and 5 years follow‐up) electronic walkways, where the middle section 5.49 m out of the total 8.7 m was measured by the electronic gait mat. In a validation study, we have demonstrated that the output from the two electronic walkways is comparable [38]. Mean preferred gait speed over two trials was used as an outcome measure in this study. Details about test protocols are described in [29].

Cut‐off criteria for key sarcopenia defining components (muscle mass, muscle strength and physical performance) were determined based on the EWGSOP2 consensus definition of sarcopenia [1] and recent normative data for the Norwegian population including the fourth wave of the Trondelag Health Study [31] and the seventh wave of the Tromsø study [30, 32] (Table 1). All cut‐off points were set at −2SD from Norwegian reference means, defining low muscle strength as grip strength < 37.0 kg (men) and < 22.2 kg (women); low muscle mass as an SMI < 9.31 kg/m^2^ (men) and < 7.45 kg/m^2^ (women); and low physical performance as gait speed < 0.88 m/s (men) and < 0.85 m/s (women). Clinically defined sarcopenia [1], was divided into ‘healthy’ (≥ cut‐offs on all defining components), ‘probable sarcopenia’ (≤ cut‐off for grip strength only), ‘sarcopenia’ (≤ cut‐off for grip strength and SMI) and ‘severe sarcopenia’ (≤ cut‐off for grip strength, SMI and gait speed).

**TABLE 1:** Criteria used to define sarcopenia according to strength, muscle mass, and physical performance.

Sarcopenia defining variables cut‐off criteria	Males	Females	Author and publication
**Low strength**			
Grip strength measured by dynamometer (kg) < −2.0 SD in healthy Norwegian adults aged 40–44 years (231 men and 281 women) from the seventh wave of the Tromsø study.	< 37.0	< 22.2	Johansson et al. 2023^a^
**Low muscle mass**			
SMI calculated from measured appendicular skeletal muscle mass from BIA, divided by height squared (kg/m^2^) < −2.0 SD in healthy Norwegian adults (Trondelag County) aged 20–39 years (4698 women and 3648 men) from the fourth wave of the Trondelag Health Study (HUNT4).	< 9.31	< 7.45	Berg et al.2024^b^
**Low physical performance**			
Measured as average velocity during a 4‐m walk test (m/s) < −2.0 SD in home‐dwelling Norwegian adults aged 40–44 years (213 men and 263 women) from the seventh wave of the Tromsø study	< 0.88	< 0.85	Bergland et al. 2019^c^

^a^ Johansson J, Grimsgaard S, Strand BH, Sayer AA, Cooper R. Comparing associations of handgrip strength and chair stand performance with all‐cause mortality‐implications for defining probable sarcopenia: The Tromsø Study 2015–2020. *BMC Med*. Nov 20, 2023;21(1):451. doi:10.1186/s12916‐023‐03172‐3.

^b^ Berg J, Nauman J, Wisløff U. Normative values for body composition in 22,191 healthy Norwegian adults 20‐99 years: The HUNT4 study. *Prog Cardiovasc Dis*. Jul–Aug 2024;85:82–92. doi:10.1016/j.pcad.2024.06.002.

^c^ Bergland A, Strand BH. Norwegian reference values for the Short Physical Performance Battery (SPPB): The Tromsø Study. *BMC Geriatr*. Aug 8 2019;19(1):216. doi:10.1186/s12877‐019‐1234‐8.

### 2.4 Statistical Analysis

Mean ± standard deviation (SD) was used to describe continuous variables and counts and percentages to describe categorical variables. To test the primary hypothesis, intention to treat linear mixed model analysis was used with the defining components of sarcopenia (grip strength, muscle mass and gait speed) entered separately as dependent variables. Fixed effects of time (0, 1, 3 and 5 years) and time × group interaction were included, and adjusted for gender (male/female), co‐habitation status (yes/no) and age at baseline [28]. *Participant* was included as a random effect. The normality of residuals was checked by visual inspection of QQ plots. As nonnormally distributed residuals were observed, we applied bootstrapping with 5000 bootstrap samples for all three components of sarcopenia.

To test the secondary hypothesis, ordinal logistic mixed model regression was used with the dependent variable defined as healthy, probable sarcopenia, sarcopenia and severe sarcopenia. This analysis disregarded the 1‐year data as physical performance (gait speed) was not tested, and all components were necessary to model clinically defined sarcopenia.

The original sample size calculation for the Generation 100 study is described in the protocol article for the main study, based on expected mortality rates [29]. We considered a *p* value of < 0.05 to indicate statistical significance. However, as we investigated multiple endpoints including all time points for all three components (*n* = 16), *p* values for the main hypothesis were adjusted using the Benjamini–Hochberg correction [39, 40]. Statistical analyses were performed in STATA 18.0 and RStudio.

## 3 Results

### 3.1 Baseline Characteristics

Participants (*n* = 1567) had a mean age of 72 years, with approximately 50% women, ~50% with higher education, 86%–90% reporting good or very good health and 73%–76% meeting Norwegian physical activity guidelines. Group‐specific baseline characteristics are shown in Table 2, and further cohort details are provided elsewhere [28, 29].

**TABLE 2:** Baseline characteristics of participants (*n* = 1567) assigned to control group (CON), moderate exercise (MICT) and high intensity exercise (HIIT) groups.

	CON	MICT	HIIT
Total number of participants	780	387	400
Age (years), mean (SD)	72.4 (2.0)	72.4 (2.0)	72.5(2.1)
Women, *n* (%)	401 (51.4)	199 (51.4)	190 (47.5)
Living alone, *n* (%)	184 (24.3)	107 (28.5)	91 (23.6)
Higher education^a^, *n* (%)	373 (49.5)	192 (51.9)	196 (50.9)
Self‐reported good health, *n* (%)^b^	649 (87.3)	314 (86.1)	340 (89)
Body mass index^c^, mean (SD)	25.9 (3.4)	25.9 (3.7)	26.2 (3.7)
Skeletal muscle index (SMI)^d^, kg/m^2^	9.7 (1.3)	9.8 (1.2)	9.9 (1.3)
Adhering to PA guidelines^e^	528 (73)	271(76)	282 (76)

Abbreviations: ADL = activities of daily living, kg = kilograms, m = meter, *n* = number of participants, SD = standard deviation.

^a^ College or university.

^b^ Good or very good.

^c^ Defined as height^2^/kg × 100.

^d^ Skeletal muscle index, SMI = total skeletal muscle mass (kg) divided by height (m^2^).

^e^ Thirty minutes of physical activity (PA) daily.

### 3.2 Time‐Dependent Changes in Sarcopenia Components

Absolute values are presented in Table 3, and adjusted estimates from the linear mixed models are shown in Table 4.

**TABLE 3:** Descriptive statistics for all components of sarcopenia (grip strength, SMI and gait speed) for all groups (CON, MICT and HIIT) for all years (BL, 1, 3, 5) by gender (male/female).

		CON					MICT						HIIT					
	Total		Women		Men		Total		Women		Men		Total		Women		Men	
	*n*	Mean (SD)	*n*	Mean (SD)	*n*	Mean (SD)	*n*	Mean (SD)	*n*	Mean (SD)	*n*	Mean (SD)	*n*	Mean (SD)	*n*	Mean (SD)	*n*	Mean (SD)
**Hand grip strength, measured by dynamometer (kg)**																		
Baseline	764	34.02 (10.79)	396	25.64 (5.14)	368	43.03 (7.52)	371	33.83 (10.58)	196	25.70 (4.82)	175	42.95 (7.34)	390	35.83 (11.25)	186	26.21 (5.58)	204	44.60 (7.23)
Year 1	607	33.37 (11.10)	322	24.95 (4.97)	285	42.89 (7.97)	280	34.47 (10.93)	138	25,50 (5.39)	142	43,19 (7.28)	279	36.27 (11.48)	131	26.45 (5.30)	148	44.95 (7.90)
Year 3	514	28.69 (10.71)	260	20.19 (4.70)	254	37.39 (7.74)	241	28.89 (10.03)	120	20.62 (4.37)	121	37.12 (6.74)	246	30.14 (10.95)	114	21.18 (4.95)	132	37.88 (8.53)
Year 5	489	27.74 (10.73)	243	19.41 (4.84)	246	35.96 (8.34)	235	27.52 (10.59)	116	19.22 (4.67)	119	35,61 (8.20)	224	29.24 (11.52)	104	20.00 (5.98)	120	37.25 (8.85)
**SMI, measured as appendicular skeletal muscle mass from BIA, divided by square height (kg/m** ^**2**^ **)**																		
Baseline	760	9.74 (1.27)	393	8.78 (0.68)	367	10.77 (0.90)	375	9.83 (1.25)	194	8.94 (0.75)	181	10.78 (0.94)	388	9.90 (1.30)	186	8.85 (0.79)	202	10.86 (0.86)
Year 1	639	9.68 (1.24)	338	8.74 (0.73)	301	10.73 (0.78)	297	9.82 (1.20)	145	8.93 (0.75)	152	10.68 (0.88)	287	9.85 (1.26)	132	8.78 (0.70)	155	10.76 (0.84)
Year 3	541	9.61 (1.22)	273	8.71 (0.76)	268	10.53 (0.87)	249	9.74 (1.26)	121	8.80 (0.75)	128	10.63 (0.96)	254	9.72 (1.20)	119	8.72 (0.73)	135	10.60 (0.76)
Year 5	494	9.55 (1.20)	250	8.65 (0.73)	244	10.47 (0.83)	246	9.65 (1.17)	120	8.79 (0.77)	126	10.47 (0.85)	227	9.59 (1.16)	108	8.67 (0.72)	119	10.42 (0.80)
**Preferred gait speed, measured as average velocity during a 4**‐**m walk test (m/s)**																		
Baseline	766	1.31 (0.20)	396	1.30 (0.20)	370	1.32 (0.20)	373	1.32 (0.20)	195	1.31 (0.19)	178	1.34 (0.20)	396	1.30 (0.21)	189	1.29 (0.22)	207	1.33 (0.19)
Year 1^a^																		
Year 3	521	1.30 (0.21)	267	1.28 (0.22)	254	1.32 (0.21)	240	1.30 (0.22)	120	1.29 (0.21)	120	1.31 (0.24)	248	1.34 (0.20)	117	1.32 (0.21)	131	1.35 (0.18)
Year 5	499	1.27 (0.21)	244	1.26 (0.22)	255	1.27 (0.21)	233	1.24 (0.20)	114	1.24 (0.24)	119	1.24 (0.20)	230	1.29 (0.21)	104	1.27 (0.23)	126	1.31 (0.20)

Abbreviations: BIA = bioimpedance analysis, CON = control, HIIT = high intensity training, kg = kilograms, kg/m^2^ = kilograms per square meter, MICT = moderate intensity training, m/s = meter per second, SD = standard deviation, SMI = skeletal muscle index.

^a^ Year 1 not tested for gait speed due to lack of testing personnel.

**TABLE 4:** Estimated differences (95% CI) in change from baseline to 1, 3 and 5 years between groups (MICT vs. CON and HIIT vs. CON) for the defining sarcopenia components; grip strength, SMI and gait speed.

	CON	MICT	HIIT	MICT vs. CON			HIIT vs. CON		
	Δ from baseline^a^	Δ from baseline^a^	Δ from baseline^a^	Estimated Δ (95% CI)	*p* ^1^	*p* ^2^	Estimated (95% CI)	*p* ^1^	*p* ^2^
**Grip strength, kilograms**									
Year 1	−0.85	−0.23	0.13	0.62 (0.10, 1.13)	0.019	0.061	0.98 (0.48, 1.47)	< 0.001	< 0.001
Year 3	−5.95	−5.91	−6.06	0.05 (−0.46, 0.56)	0.851	0.959	−0.10 (−0.70, 0.50)	0.736	0.959
Year 5	−7.12	−7.32	−6.81	−0.20 (−0.89, 0.48)	0.562	0.899	0.31 (−0.31, 0.92)	0.327	0.747
**Skeletal muscle index, kg/m** ^**2**^									
Year 1	−0.06	−0.06	−0.08	−0.001 (−0.05, 0.04)	0.948	0.959	−0.01 (−0.06, 0.03)	0.525	0.899
Year 3	−0.16	−0.16	−0.18	−0.002 (−0.07, 0.06)	0.959	0.959	−0.02 (−0.08, 0.04)	0.516	0.899
Year 5	−0.24	−0.24	−0.31	0.005 (−0.05, 0.06)	0.869	0.959	−0.07 (−0.12, 0.01)	0.018	0.060
**Gait speed, m/s**									
Year 1^b^	—	—	—	—	—	—	—	—	—
Year 3	−0.03	−0.04	0.01	−0.01 (−0.03, 0.02)	0.678	0.959	0.03 (0.01, 0.06)	0.002	0.016
Year 5	−0.07	−0.10	−0.04	−0.03 (−0.06, −0.01)	0.011	0.059	0.02 (−0.004, 0.05)	0.093	0.248

*Note:* Grip strength, measured by dynamometer (kg); SMI, measured as appendicular skeletal muscle mass from BIA, divided by height squared (kg/m^2^), gait speed, measured as, average velocity during a 4‐m walk test (m/s), *p* ^1^ = after bootstrap; *p* ^2^ = after correction for multiple testing of *p* values at all time points for all three components (*n* = 16) (Benjamini–Hochberg).

Abbreviations: BIA = bioimpedance analysis; CON = control, HIIT = high intensity training, kg = kilograms, kg/m^2^ = kilograms per square meter, MICT = moderate intensity training, m/s = meter per second, SD = standard deviation, SMI = skeletal muscle index.

^a^ Based on model adjusted estimates (sex, co‐habitation and age at baseline).

^b^ Year 1 not tested for gait speed due to lack of test personnel.

Overall, grip strength declined in all groups over time, with HIIT showing a modest short‐term improvement at Year 1. From baseline to Year 1, CON and MICT declined (−0.85 and −0.23 kg), while HIIT increased slightly (+0.13 kg), resulting in a significant difference for HIIT versus CON (0.98 kg; *p* < 0.001). At Years 3 and 5, all groups showed similar declines (approximately −6 and −7 kg from baseline), with no significant between‐group differences.

SMI decreased gradually in all groups across the 5 years. Declines were comparable between groups at each time point, although HIIT showed a slightly larger reduction than CON at Year 5 (−0.31 vs. −0.24 kg/m^2^), without significant between‐group differences.

Patterns of change in gait speed showed small differences between the three groups. At Year 3, CON and MICT had slight reductions (−0.03 and −0.04 m/s), while HIIT increased by +0.01 m/s (HIIT vs. CON difference 0.03 m/s; *p* = 0.016). At Year 5, all groups showed declines from baseline (−0.07 to −0.10 m/s), with no significant between‐group differences.

### 3.3 Effects on Clinically Defined Sarcopenia

Results from the ordinal logistic mixed model are shown in Table 5. At baseline, most participants were classified as ‘healthy’ (~80%), with approximately 20% having ‘probable sarcopenia’, and < 2% meeting criteria for sarcopenia or severe sarcopenia. At Year 3, the prevalence of probable sarcopenia increased across all groups (CON 52%, MICT 55%, HIIT 48%). At Year 5, MICT had the highest proportion (62%), followed by CON (57%) and HIIT (51%). The odds of developing probable sarcopenia were significantly lower in HIIT compared with CON at both Year 3 (OR = 0.51; *p* = 0.018) and Year 5 (OR = 0.47; *p* = 0.009).

**TABLE 5:** Descriptives (counts and percentages) and odds ratios for the four‐category model classifying clinically defined sarcopenia between groups (MICT vs. CON and HIIT vs. CON) at Years 3 and 5. Odds ratios are presented with 95% CI and *p* values.

		CON, *n* = 780	MICT, *n* = 387	HIIT, *n =* 400	MICT vs. CON		HIIT vs. CON	
Year	Category	Frequency (%)	Frequency (%)	Frequency (%)	ORs (95% CI)	*p*	ORs (95% CI)	*p*
0	Healthy	594 (78.1)	295 (79.5)	312 (80.4)	—	—	—	—
	Probable Sarcopenia	155 (20.4)	68 (18.3)	73 (18.8)				
	Sarcopenia	11(1.4)	6 (1.6)	3 (0.8)				
	Severe Sarcopenia	1 (0.1)	2 (0.5)	0				
	Missing	19 (19.4)	16 (4.1)	12 (3.0)				
3	Healthy	211 (41.6)	108 (45.2)	121 (49.6)	0.88 (0.51 to 1.53)	0.648	0.51 (0.29 to 0.89)	0.018
	Probable Sarcopenia	277 (54.6)	125 (52.3)	117 (48.0)				
	Sarcopenia	18 (3.6)	5 (2.1)	6 (2.5)				
	Severe Sarcopenia	1 (0.2)	1 (0.4)	0				
	Missing	178 (26.0)	72 (23.2)	52 (17.6)				
5	Healthy	178 (36.9)	78 (33.8)	98 (44.1)	1.26 (0.72 to 2.21)	0.414	0.47 (0.26 to 0.83)	0.009
	Probable Sarcopenia	274 (56.8)	142 (61.5)	114 (51.4)				
	Sarcopenia	27 (5.6)	9 (3.9)	10 (4.5)				
	Severe Sarcopenia	3 (0.6)	2 (0.9)	0				
	Missing	142 (22.8)	55 (19.2)	46 (17.1)				

*Note:* Categories: Healthy = above the cut‐offs on all sarcopenia components, probable sarcopenia = equal to or below cut‐off grip strength, sarcopenia = equal to or below cut‐off grip strength and SMI, and severe sarcopenia = equal to or below cut‐off grip strength, SMI and gait speed.

Abbreviations: CI = confidence intervals, CON = control, HIIT = high intensity training, MICT = moderate intensity training, ORs = odds ratios.

## 4 Discussion

In this study of relatively healthy and active older adults from the Generation 100 cohort, we demonstrated a possible prevention effect over 5 years for high intensity aerobic interval training (HIIT) on key components of sarcopenia—grip strength, SMI and gait speed—and clinically defined sarcopenia—‘probable sarcopenia’, ‘sarcopenia’ and ‘severe sarcopenia’. Although the participants subsequently aged during this study, our findings show that those randomized to HIIT experienced a significantly attenuated decline compared with the control group (CON) in Years 1 and 3. This pattern was not observed in the moderate‐intensity continuous training (MICT) group.

Although comparable studies are limited and mainly focus on beneficial muscle changes at the cellular level [26, 27], our findings extend this by demonstrating functional adaptations of relevance to prevention of sarcopenia in older adults. HIIT, when performed as vigorous uphill walking in our study, likely engaged quite vigorous body movements, including use of trekking poles, and thus contributed to greater upper body strength gains in this group. We have previously reported that adherence to HIIT was highest in the first year of intervention [28] possibly explaining why the benefits observed were not sustained over time. After Year 1, declines in grip strength were larger than 6.5 kg in all three groups, which is considered a clinically relevant decline [41]. Long‐term adherence is a challenge, and strategies to support sustained engagement are therefore essential [42]. Even so, walking‐based programmes, including HIIT, remain highly valuable for independently living older adults, as they can be easily performed outdoors, require minimal equipment, supervision and cost and are associated with increased overall physical activity and better self‐reported health [26, 43].

At Year 3, we found a beneficial effect of HIIT—but not MICT on gait speed. The between‐group difference (0.03 m/s) falls within the threshold for clinically meaningful change in community‐dwelling older adults [44]. In a highly fit population of older adults, even modest gait speed changes likely represent early stages of decline in neuromuscular capacity, given that individuals near their functional ceiling show proportionally smaller but clinically relevant changes [39]. This suggests that HIIT may possibly help preserve functional capacity long term.

In contrast to the strength and gait speed findings, we observed no between‐group differences in SMI, with all groups exhibiting similar declines over time (0.06–0.08 kg/m^2^ at Year 1; 0.16–0.18 kg/m^2^ at Year 3; 0.24–0.31 kg/m^2^ at Year 5). These declines fall within or below expected age‐related trajectories [40]. During the intervention period, SMI declined by 0.5%–4%, which is lower than the 5%–10% loss for older adults [41], suggesting that the loss of muscle mass occurred similarly across groups and may reflect a general aging effect rather than a response to training intensity.

Our findings for clinically defined sarcopenia extend this picture. The HIIT group had significantly lower odds of developing both ‘probable sarcopenia’ and ‘sarcopenia’ at Years 3 and 5 relative to CON. These findings suggest that functional indicators of sarcopenia respond more readily to HIIT than structural measures like SMI. Given that grip strength largely determines classification into ‘probable sarcopenia’, our results further highlight its value as a central marker in clinical screening [1, 11]. Very few participants in this study developed ‘severe sarcopenia’ (< 1%), which aligns with the high baseline gait speed (~1.30 m/s) in this cohort, indicating a highly functional sample. Our results may reflect that small but meaningful changes in physical function are easier to detect in sarcopenia components that are more sensitive to exercise intensity, such as grip strength and gait speed, than in SMI.

This study has limitations that may influence the interpretation of results. Firstly, we have based our analysis on secondary outcomes from a RCT, which might have biased effect estimates. Though, we reduced the risk by following a predefined statistical plan consistent with the primary outcomes—using intention to treat linear mixed models—and applied a rather strict correction on our estimates. Attrition may have introduced selection bias in this study. Our mixed models mitigate this bias but we cannot fully eliminate this possibility.

A key explanation for the null effect of MICT in this study is that the CON group did not behave as a sedentary reference group. As previously reported [27], the CON group had a high level of activity throughout the study, suggesting they performed at an activity level between MICT and HIIT. The contrast required to detect differential effects between the CON and MICT groups in this study might have been limited by this.

Another methodological limitation is generalizability. Our participants were relatively healthy older adults, living in Norway and their high functional level may not reflect frailer or more clinically diverse populations. We strengthened our classification of sarcopenia by refining the EWGSOP2 cut‐offs for these outcomes with regional normative references of the healthy young Norwegian population [30, 31, 32]. To our knowledge, this is the first study to apply and adapt the current diagnostic criteria for sarcopenia in a clinical trial setting in Norway, and research to refine and unify these criteria is necessary to design trials for prevention and treatment of sarcopenia and further research needs to validate them in other populations of older adults.

## 5 Conclusion

In this large‐scale, 5‐year clinical trial of relative healthy older adults participating in the Generation 100 study in Norway, we found that HIIT appears to preserve grip strength and gait speed, two core determinants of sarcopenia in older adults, more effectively than MICT and following the national guidelines for PA. This study underscores the importance of integrating HIIT in strategies for delaying or preventing functional decline alongside supportive strategies to secure long‐term adherence in diverse populations of older adults.

## Ethics Statement

The study was approved by the Regional Committee for Medical Research Ethics (REC South‐East B; REK 2012/381 B). All participants gave their informed consent prior to their inclusion in the study. The study was registered in ClinicalTrials.gov in August 2012 (NCT01666340).

## Conflicts of Interest

The authors including Guro Birgitte Stene, Dorthe Stensvold, Trude Rakel Balstad, Maria Jose Lequerica, Stian Lydersen, Ulrik Wisløff and Jorunn Lægdheim Helbostad declare that they have no conflicts of interest. The Generation 100 Study was supported by the Research Council of Norway; The K.G. Jebsen Foundation for Medical Research, Norway; Norwegian University of Science and Technology (NTNU); Central Norway Regional Health Authority; St Olavs hospital, Trondheim, Norway; and the National Association for Public Health, Norway. The funding organizations had no role in the design and conduct of the study; in the collection, analysis and interpretation of the data; or in the preparation, review or approval of the manuscript.
